# *Desmodesmus subspicatus* co-cultured with microcystin producing (PCC 7806) and the non-producing (PCC 7005) strains of *Microcystis aeruginosa*

**DOI:** 10.1007/s10646-019-02082-6

**Published:** 2019-07-27

**Authors:** Azam Omidi, Maranda Esterhuizen-Londt, Stephan Pflugmacher

**Affiliations:** 1 Technische Universität Berlin, Chair Ecological Impact Research and Ecotoxicology, Ernst-Reuter-Platz 1, 10587 Berlin, Germany; 2 University of Helsinki, Aquatic Ecotoxicology in an Urban Environment, Ecosystems and Environment Research Programme, Faculty of Biological and Environmental Sciences, Niemenkatu 73, 15140 Lahti, Finland; 3 Korean Institute of Science and Technology Europe (KIST), Joint laboratory of Applied Ecotoxicology, Campus E7 1, 66123 Saarbrücken, Germany; 4 Helsinki Institute of Sustainability (HELSUS), Fabianinkatu 33, 00014 Helsinki, Finland

**Keywords:** Co-cultivation, *D. subspicatus*, *M. aeruginosa*, MC-LR, Interspecies interactions

## Abstract

Although microcystins (MCs) are the most commonly studied cyanotoxins, their significance to the producing organisms remains unclear. MCs are known as endotoxins, but they can be found in the surrounding environment due to cell lysis, designated as extracellular MCs. In the present study, the interactions between MC producing and the non-producing strains of *Microcystis aeruginosa*, PCC 7806 and PCC 7005, respectively, and a green alga, *Desmodesmus subspicatus*, were studied to better understand the probable ecological importance of MCs at the collapse phase of cyanobacterial blooms. We applied a dialysis co-cultivation system where *M. aeruginosa* was grown inside dialysis tubing for one month. Then, *D. subspicatus* was added to the culture system on the outside of the membrane. Consequently, the growth of *D. subspicatus* and MC contents were measured over a 14-day co-exposure period. The results showed that *Microcystis* negatively affected the green alga as the growth of *D. subspicatus* was significantly inhibited in co-cultivation with both the MC-producing and -deficient strains. However, the inhibitory effect of the MC-producing strain was greater and observed earlier compared to the MC-deficient strain. Thus, MCs might be considered as an assistant factor that, in combination with other secondary metabolites of *Microcystis*, reinforce the ability to outcompete co-existing species.

## Introduction

In recent decades, the increasing occurrence of cyanobacterial blooms in water bodies throughout the world has raised concerns (Buratti et al. 2017; Svirčev et al. 2017). Moreover, global warming and eutrophication have increased the occurrence of cyanobacterial blooms with a shift toward more toxic populations (Dziallas and Grossart 2011; Scholz et al. 2017). Cyanobacteria are known to produce toxic secondary metabolites designated as cyanotoxins which have undesirable effects on humans, animals, and aquatic organisms (Catherine et al. 2013; Zanchett and Oliveira-Filho 2013; Lévesque et al. 2014). Among the cyanotoxins, microcystins (MCs) are the most commonly studied. However, past studies have mainly focused on the toxicity of MCs while their importance to the producing organisms is still unknown. Recently, there has been a considerable growing interest in understanding the physiological and ecological importance of MCs to the producers beyond their toxicity.

MCs are produced by many species of cyanobacteria, however the most common bloom-forming species is *Microcystis aeruginosa* (Carmichael 1992). They are high-cost products which are synthesised non-ribosomally *via* a complex multifunctional enzyme, MC synthetase (Nishizawa et al. 2000; Welker and Von Döhren 2006). It is still uncertain why producers pay such a high energy price for the synthesis of MCs. A study by Christiansen et al. (2008) revealed that non-toxic strains could produce other non-ribosomal peptides, but not MC variants due to the partial or total lack of the MC synthetase genes, *mcy* gene cluster. MC-deficient strains, therefore, can be useful tools in competitive studies with toxic strains, to clarify the importance of MCs for the producing species. Previous studies demonstrated that MC-producing strains gain advantages from MC production over non-toxic subpopulations for better environmental adaptations under low or high light irradiation (Renaud et al. 2011), C-limited conditions (Jähnichen et al. 2007; Zhang et al. 2012), and elevated water temperature (Dziallas and Grossart 2011).

Recent studies have suggested some ecological and physiological functions for MCs; varying from the interference in photosynthesis and nutrient metabolism to quorum sensing, iron uptake, recruitment, defence against grazers, and allelopathic interactions (Omidi et al. 2017). The toxin-related interspecies interactions between *Microcystis* and other co-existing organisms such as the members of the phytoplankton community may further elucidate the ecological importance of MCs.

Toxin production by *M. aeruginosa* has been suggested to be a continuous process that starts in the early logarithmic phase to the beginning of the stationary phase (Lyck 2004; Jähnichen et al. 2008). MCs can be enclosed within the cells (intracellular MCs) or released into surrounding water (extracellular MCs) (Sivonen and Jones 1999) at different growth stages and under various environmental or physiological conditions, by cell lysis or leakage of intracellular MCs (Rapala et al. 1997; Leflaive and Ten-Hage 2007). Varying levels of MCs have been detected in the water bodies ranging from <3 µg L^−1^ across Europe to 19,500 µg L^−1^ in water samples from Japan (Turner et al. 2018). During senescence of scums or very dense cyanobacterial blooms, high concentrations of MC reaching up to 25,000 µg L^−1^ have been reported (Sivonen and Jones 1999; World Health Organization 2003).

When blooms collapse due to artificial (mechanical or chemical) or natural processes, the cellular material containing high concentrations of toxins are released into the environments in a short period. In nature, the toxin concentrations do not stay at these high level indefinitely due to rapid dilution in the water column and degradation of MCs by light or certain species of bacteria (Christoffersen et al. 2002; Gągała and Mankiewicz-Boczek 2012). However, before this happens, aquatic biota is constantly exposed to MCs for days (Jones and Orr 1994).

In aquatic habitats, the cyanobacterial blooms are frequently associated with green algae, another member of the phytoplankton community (Sedmak and Kosi 1998; Paerl et al. 2001). The phytoplankton community, contain other members such as diatoms, dinoflagellates, and a diverse group of algae as well (Reynolds 2006). The species composition of phytoplankton communities are dynamic and change in seasonal cycles, a phenomenon called seasonal successions where according to the seasonal pattern the species dominate the phytoplankton community in successive waves (Reynolds 1980; Sedmak and Kosi 1998; Chen et al. 2003; El Herry et al. 2008). Past studies have shown that not only the abiotic environmental conditions, but also the biological factors such as interspecies interferences, influenced the seasonal fluctuations of the algal populations in the phytoplankton community (Vardi et al. 2002; Chen et al. 2003; Legrand et al. 2003; Granéli and Hansen 2006; Leão et al. 2009; Zhang et al. 2015).

Therefore, the present study aimed to understand the influence and possible advantage of toxin production better, i.e. if it provided a competitive advantage to the MC-producing strain of *M. aeruginosa*. A comparative study between toxic (MC-producing) and non-toxic (MC-deficient) strains of *M. aeruginosa*, and *Desmodesmus subspicatus*, a freshwater green alga, was performed in a co-exposure system to explore the probable importance of the cyanobacterial bioactive metabolites, especially MCs, when the bloom material starts to lyse, and a high concentration of toxin is released into the surrounding environment.

## Materials and methods

### Organisms and culture conditions

**Fig. 1:**
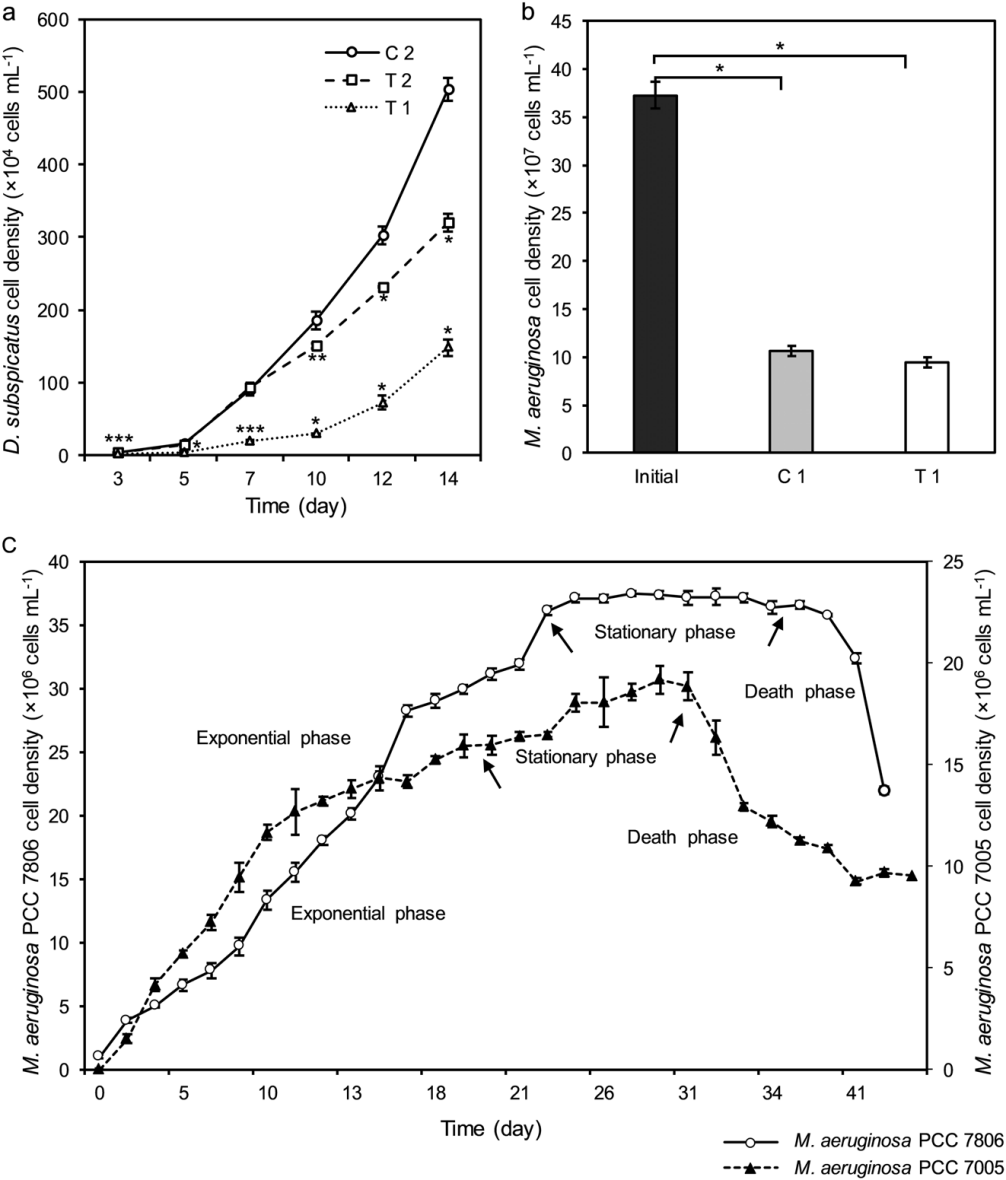
The growth of *D. subspicatus* SAG 86.81 on the outside of the dialysis tubing in co-cultivation with *M. aeruginosa***a**, the density of *M. aeruginosa* 7806 inside of the dialysis tubing in co-cultivation with *D. subspicatus***b**, and the growth curves of *M. aeruginosa* PCC 7806 and PCC 7005 **c** (initial, 1-month-old monoculture of *M. aeruginosa* PCC 7806; C 1: control 1, 6 weeks old monoculture of *M. aeruginosa* PCC 7806; C 2: control 2, monoculture of *D. subspicatus*; T 1: treatment 1, co-culture of *M. aeruginosa* PCC 7806 with *D. subspicatus*; T 2: treatment 2, co-culture of *M. aeruginosa* PCC 7005 with *D. subspicatus*). Data represent mean values ± standard deviation (*n* = 3). Significant differences observed at *p*-values of *p < 0.001, **p ≤ 0.01 and ***p < 0.05

Axenic cultures of *M. aeruginosa* PCC 7806 and *M. aeruginosa* PCC 7005 were obtained from the Pasteur Culture Collection of Cyanobacteria (PCC), Paris, France. The green alga, *D.subspicatus* SAG 86.81 (formerly *Scenedesmus subspicatus*), was provided by the SAG Culture Collection of Algae (Sammlung von Algenkulturen), University of Göttingen, Germany. The species were grown in BG-11 liquid medium (Rippka et al. 1979) at 24 ± 1 °C under an illumination intensity of 2220 lm m^−2^, provided by cool white fluorescent irradiation, with a 12:12 light: dark interval. The growth phases of *M. aeruginosa* PCC 7806 and PCC 7005 were studied and documented prior to experimentation (Fig. 1c).

### Co-cultivation experimental design

The dialysis membranes with a molecular weight cut-off of 12–14 kDa and diameter of 29 mm (Spectra/ Por®, Spectrum Laboratories, USA) were used to separate the cultures. The membranes were cut into 30 cm lengths and soaked in distilled water for 15 min. Then, they were incubated in 10 mM sodium bicarbonate (NaHCO_3_) for 30 min at 80 °C and soaked in 10 mM Na_2_EDTA for 30 min. The wide-open end of a glass pasture pipette was used to hold the dialysis tube and to transfer the *Microcystis* inoculum into the tubing. One end of the membrane was tightly tied to the pasture pipette with a piece of string, as an open end to the latter injection of the cells into the dialysis tubing. Another end was tied closed by a knot, then fastened to the glass pipette with the string. The prepared tubings were autoclaved for 10 min at 121 °C and placed aseptically in a 250 mL glass bottle containing 180 mL sterile BG-11 medium. The dialysis tubings were filled with 15 mL fresh sterilized BG-11 medium and *Microcystis* inoculum in 5 mL fresh BG-11 medium was injected into the dialysis tubing using a sterile syringe.

Monocultures of *M. aeruginosa* in the dialysis tubing, containing 20 mL fresh, sterilised BG-11 medium, nine replicates of *M. aeruginosa* PCC 7806 (MC-producing strain), and three replicates of *M. aeruginosa* PCC 7005 (MC-deficient strain), were prepared each at the initial cell density of 1 × 10^7^ cells mL^−1^. Then, the groups of toxic and non-toxic independent replicates were grown for 1 month before commencing with the co-cultivation experiments. Every 5 days, 5 mL of fresh sterilized BG-11 medium was added out of the membrane.

After 1 month, monocultures of the toxic strain (triplicate) was harvested as ‘initial' to measure the initial concentrations of the intracellular and extracellular MC-LR before the introduction of the green algae into the culture system.

The co-cultivation experiments then commenced, i.e. they were performed using 1-month-old monocultures of toxic and non-toxic *Microcystis* (six and three replicates, respectively). *D. subspicatus* at the initial density of 1 × 10^4^ cells mL^−1^ was inoculated on the outside of the membrane into the bottle containing the 1-month-old *Microcystis* inside the dialysis membrane. As a control for the growth of the green alga, *D. subspicatus* was cultured in the bottle containing a dialysis tubing that was filled only with BG-11 medium without *M. aeruginosa*. The prepared cultures include 1-month-old monoculture of toxic *Microcystis* (control 1: C 1), co-cultures of green alga with the toxic (treatment 1: T 1) and non-toxic (treatment 2: T 2) strains of *Microcystis*, and monoculture of green alga (control 2: C 2), were kept on a shaker at 75 rpm under the same conditions as described for the unialgal cultures for 14 days.

### Growth measurement of *D. subspicatus* and *M. aeruginosa* PCC 7806

Every 2 or 3 days, 1 mL of sample was taken from the outside of the dialysis tubing from each replicate of monocultures (C 2) and co-cultures of the green alga with toxic and non-toxic strains of *M. aeruginosa* (T 1 and T 2, respectively). Then, cells of *D. subspicatus* were counted using bright field microscopy (Olympus CH-2, Japan) and a Neubauer counting chamber (Roth, Karlsruhe, Germany).

The cell density of *M. aeruginosa* PCC 7806 was monitored in 1-month-old monoculture (initial) and after 2 weeks co-cultivation from mono- (C 1) and co-cultures of toxic *Microcystis* with green alga (T 1). A 1 mL sample was collected from the inside of dialysis tubing from each replicate. The optical density of samples (OD_750_) was measured using a spectrophotometer (UVIKON 922, France). Then, the cell numbers was calculated using the calibration curve from the OD_750_ vs cells mL^−1^.

### Extracellular MC-LR preparation

Samples from the inside (5 mL) and outside (40 mL) of the dialysis tubing were collected from the 1-month-old monoculture of toxic *Microcystis* (initial) and after 2 weeks co-cultivation from mono- (C 1) and co-cultures of toxic *Microcystis* with green alga (T 1). *Microcystis* cells were harvested and the supernatant collected from the inner membrane after centrifugation at 4000 × *g* for 30 min (4 °C). The supernatant was collected from the outer side of the membrane after centrifugation (4000 × *g*, 15 min, 4 °C). The supernatants were filtered through a 0.22 µm Whatman filter (Millipore). Solid-phase extraction (SPE) was performed using C18 SPE cartridges (Sep-Pak tC18 6 cc Vac Cartridge, 500 mg Sorbent per Cartridge, pore size 125 Å, particle size 37–55 μm, hold up volume, Waters) which was conditioned with 10 mL of 100% methanol (MeOH) and subsequently washed with 10 mL of distilled water. The cell-free supernatants were applied to the pre-conditioned cartridge and eluted with 10 mL of 100% MeOH. The eluent was dried in a concentrator plus (Eppendorf, Germany) at 30 °C, and the residue was resuspended in 1 mL of MeOH 100% and stored at −20 °C until analysis.

### Intracellular MC-LR extraction

Samples of 1-month-old monoculture of toxic *Microcystis* (initial), and 2 weeks mono- (C 1) and co-cultures of toxic *Microcystis* with green alga (T 1) were collected from the inside of the dialysis tubing and were centrifuged (4000 × g, 30 min, 4 °C). Then, the pellet was freeze-dried using an LIO-5P freeze dryer (5 pascals, Italy). The lyophilised cells (25 mg) were sonicated in an ultrasonic bath (Allpax, Germany) for 15 min in 3 cycles of 5 min. After that, intracellular MC-LR was extracted by addition of 1 mL of 70% methanol (MeOH) acidified with 0.1% trifluoroacetic acid (TFA) which was continuously shaken (Intelli-mixer, neoLab®) for 1 h. The resulting supernatant was collected after centrifugation (10,000 × *g*, 10 min, 4 °C), and the pellet was re-extracted with the same procedure. This procedure was repeated four times. Then, the combined supernatant was evaporated to dryness at 30 °C using a concentrator plus (Eppendorf, Germany) and the dried material was dissolved in 1 mL of 100% MeOH and centrifuged (20,800 × *g*, 15 min, 4 °C). The supernatant was stored at −20 °C until analysis by liquid chromatography-tandem mass spectroscopy (LC-MS/MS).

### MC-LR analysis

An Alliance 2695 UHPLC coupled to a Micromass Quattro micro™ (Waters, Milford, MA, USA) was used for determination and quantification of MC-LR. A reversed phase column Kinetex™ C18 column (2.1 × 50 mm, 2.6 μm pore size, Phenomenex) was used for chromatography. Solution A (MS-grade water containing 0.1% trifluoroacetic acid (TFA) and 5% acetonitrile (ACN)) and solution B (ACN containing 0.1% TFA) was used as the mobile phase at a flow rate of 0.2 mL min^−1^. A linear gradient elution program was applied as follows: 0 min 100% A; 3.75–7 min 35% A, and 7.8–12 min 100% A. The column oven temperature was set at 40 °C. The injection volume was 20 μL. MC-LR in the samples was identified by the retention time at 7.95 min.

The tandem mass spectroscopy, using electrospray ionization (ESI), conditions were set as follows: the spray voltage was set at 3 kV, and the cone voltage at 60 V. The capillary temperature was set at 120 °C, the desolvation gas temperature and cone gas flow-rate was set at 500 °C and 1000 L h^−1^, respectively. The collision energy was 65 and cone gas flow-rate of 100 L h^−1^. The trigger gas and the collision gas were nitrogen and Argon, respectively. Parent compounds and its fragment ions, respectively, were analysed according to their alignment at the following mass-to-charge ratio (m/z) 995.5 → 135.1.

The detection limit of MC-LR was 1 ng mL^−1^ (signal to noise S/N > 3), and the limit of quantification was set at 5 ng mL^−1^ (signal to noise the S/N > 5). The toxin content was quantified by calibrating against the standard solution of purified solid MC-LR (Enzo, Germany) in MeOH 100%.

### Statistical analysis

Statistical analyses were performed with SPSS (version 24). Data were tested for normality and homogeneity of variance using the Shapiro-Wilk test and Levene test, respectively. Differences between samples were determined using one-way analyses of variance (ANOVA) followed by Turkey HSD analysis and Student’s *t* test for the homogenous (the growth of *D. subspicatus* and *M. aeruginosa 7806*, intracellular MC-LR, and the diffusion rate of extracellular MC-LR) and heterogeneous variables (extracellular MC-LR), respectively (*p* ≤ 0.05). Data, which did not follow a normal distribution (growth of *D. subspicatus* on the 3rd day (treatments (T1 and T2), and the 7th day (treatment with *M. aeruginosa* PCC 7806 (T1)) was analysed with non-parametric tests, such as the Kruskal Wallis and Mann–Whitney-U-test.

## Results

### Inhibition of growth rate

After co-cultivation with the MC-producing *Microcystis* (treatment 1, T 1), the biomass of *D. subspicatus* was significantly decreased compared to the biomass concentration achieved in monoculture (C 2) and also co-culture with the MC-deficient strain (treatment 2, T 2) (*p* < 0.05, Fig. 1a). The results indicated a long lag phase, from day 3 to 10, in the growth of green alga co-cultivated with PCC 7806. After 10 days, the density of green alga in co-cultivation with PCC 7806, sharply levelled off to 29 × 10^4^ cells mL^−1^, 6.3 and 5.1 times less than when grown as mono- or in co-culture with the MC-deficient strain, respectively (*p* < 0.001). Afterwards, the growth of the green alga slowly increased and reached 148 × 10^4^ ± 12 × 10^4^ cells mL^−1^ on day 14, which was 3.4 times less than in monoculture and 2.16 times lower than when co-cultured with the MC-deficient strain (*p* < 0.001).

In contrast, the density of the green alga, which was co-cultivated with MC-deficient strain, *M. aeruginosa* PCC 7005, was not significantly different to the control after the first 7 days (*p* > 0.05). Afterwards, the biomass of green alga was decreased significantly and slowly fell to 320 × 10^4^ ± 13 × 10^4^ cells mL^−1^ at the end of the co-cultivation experiments on day 14 that was 1.6 times lower than the monoculture (*p* < 0.001, Fig. 1a).

The results showed that after 14 days of co-cultivation, the cell density of *M. aeruginosa* 7806 was significantly decreased in mono- (C 1) and co-cultures (T 1), compared to the 1-month-old monoculture (initial) (*p* < 0.001, Fig. 1b). Moreover, the growth of *Microcystis* in co-culture (T 1) remained unchanged, compared to the simultaneously conducted monoculture (C 1) (*p* > 0.05, Fig. 1b).

### Toxin concentration

**Fig. 2:**
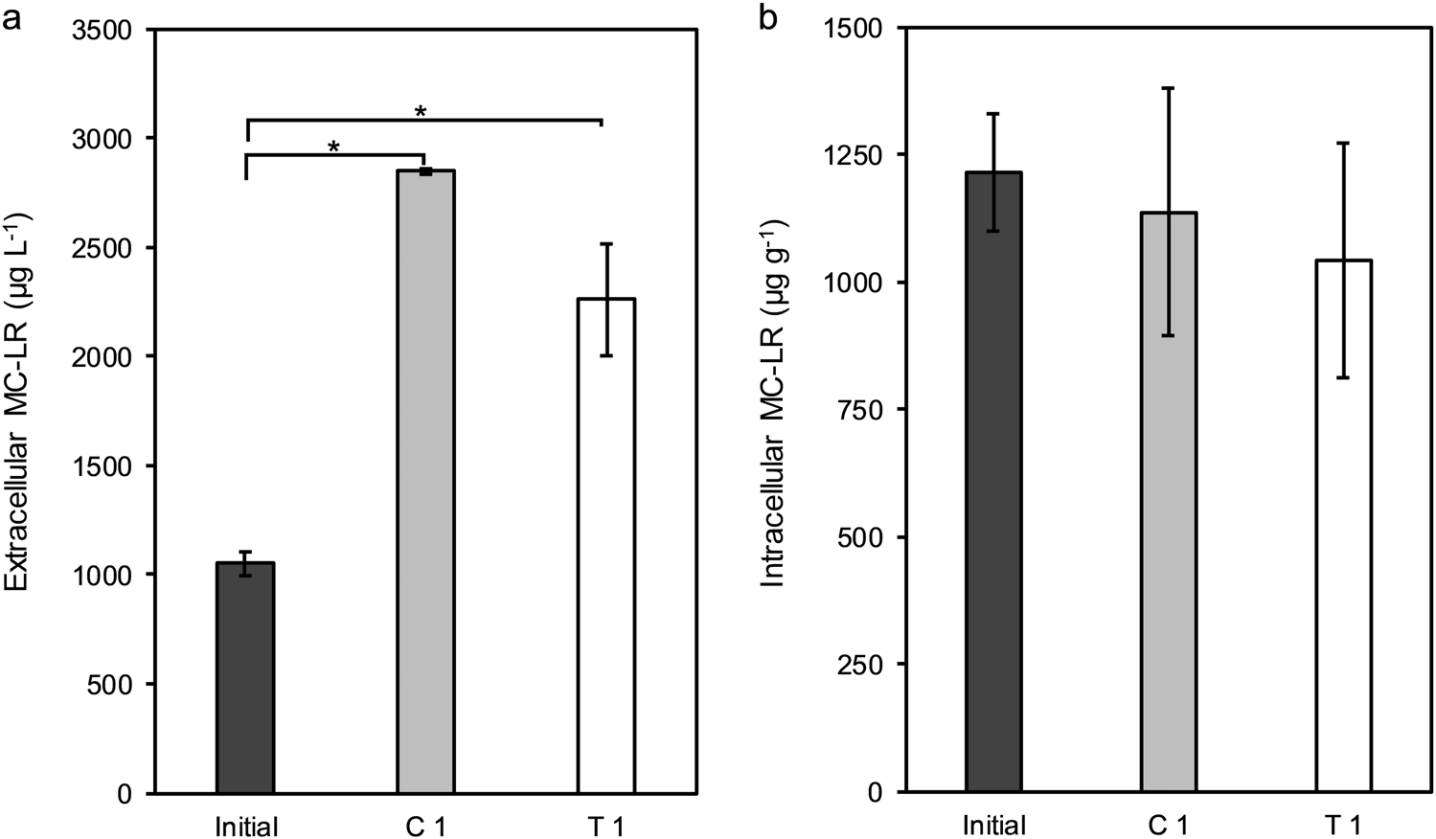
The concentration of **a** total extracellular MC-LR (µg L^−1^) and **b** intracellular MC-LR (µg g^−1^ dry weight) where initial is the monoculture of *M. aeruginosa* PCC 7806 sampled after 1 month, C 1 is the monoculture of *M. aeruginosa* PCC7806 sampled 14 days after inception of co-cultivation experiment (6 weeks), and T 1 is the co-culture of *M. aeruginosa* PCC 7806 with green alga-after 14 days. Data represent mean values ± standard deviation (*n* = 3). Asterisk indicates significant differences at a *p*-value of *p* ≤ 0.001

At the inception of the experiment in 1-month-old monoculture (initial), the total concentration of extracellular MC-LR (inside plus outside the dialysis membrane) was 1050.2 ± 51.7 µg L^−1^ (Fig. 2a) and the intracellular MC-LR concentration was 1214.5 ± 114.5 µg g^−1^ (Fig. 2b). After 2 weeks of co-cultivation, the concentration of intracellular MC-LR in mono- (C 1) and co-cultures (T 1) remained unchanged compared to the intracellular MC-LR concentration at the start of the experiment (initial) (Fig. 2b, *p* > 0.05). In contrast, the concentration of extracellular MC-LR was significantly raised both with mono- and co-cultivation (*p* ≤ 0.001, Fig. 2a). However, after 2 weeks of co-cultivation, the increased MC release in co-culture (T 1) was at the same level as the simultaneous monoculture (C 1) (*p* > 0.05, Fig. 2a). Additionally, the amount of outer membrane extracellular MC-LR that was the actual concentration at which the green alga has been exposed was not significantly different relative to the control (C 1) (*p* > 0.05, Fig. 2).

**Fig. 3:**
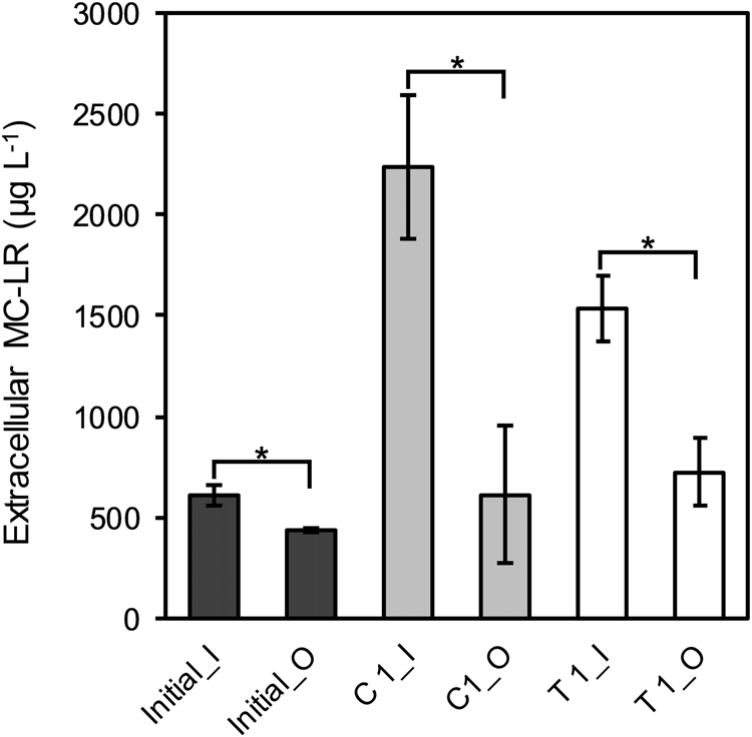
The concentration of the extracellular MC-LR (µg L^−1^) in the controls and treatment on the inside and outside of the dialysis tubing, measured after 1 month in monoculture (initial) and 6 weeks in monoculture (control 1) and co-culture (treatment 1) of toxic *M. aeruginosa* PCC 7806 (Initial: 1-month-old monoculture, C 1: control 1, T 1: treatment 1, I: in the dialysis tubing, O: out of the dialysis tubing). Data represent means ± standard deviation (*n* = 3). Asterisk indicates significant differences at a *p*-value of *p* < 0.01

**Fig. 4:**
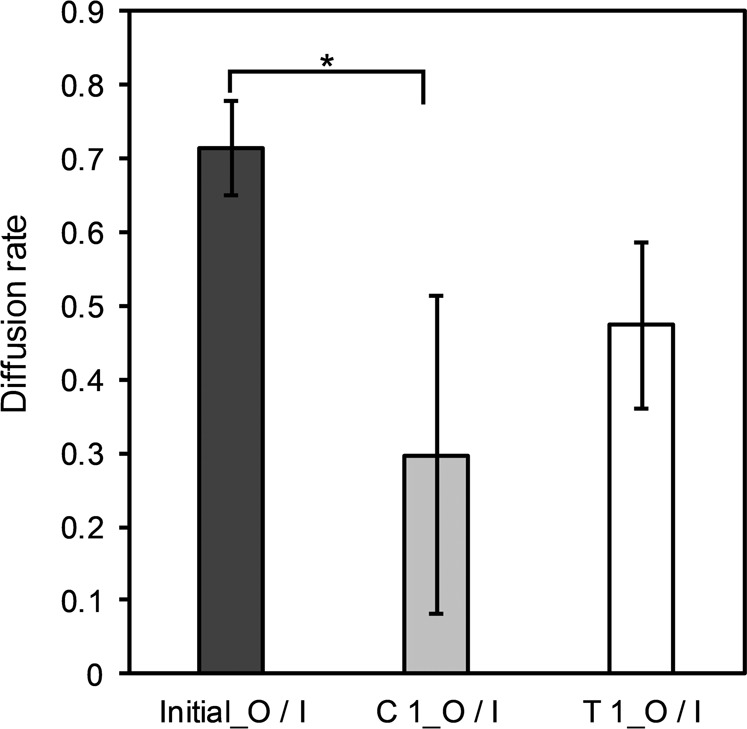
The diffusion rate of extracellular MC-LR in controls and treatment (Initial: 1-month-old monoculture of *M. aeruginosa* PCC 7806; C 1: control 1, 6 weeks old monoculture of *M. aeruginosa* PCC 7806; T 1: treatment 1, co-culture of *M. aeruginosa* PCC 7806 with *D. subspicatus*, I/O: in/out of dialysis tubing). Data represent means ± standard deviation (*n* = 3). Significant difference observed at a *p*-value of *p < 0.05

The efficiency of employing the dialysis membrane in the co-cultivation system was assessed in terms of the diffusion rate of MC-LR. This was evaluated by measuring the concentration of extracellular MC-LR at both sides of the dialysis tubing (Fig. 3). The results showed that at the start of the co-cultivation experiment with *M. aeruginosa* PCC7806 monoculture (initial), as well as after 14 days of both mono- (C 1) and co-culture (T 1), the concentration of extracellular MC-LR inside the dialysis membrane was significantly higher than the outer of the membrane (*p* < 0.01, Fig. 3). However, in the 1-month-old monoculture (initial) where the lowest concentration of extracellular MC-LR was measured, the highest diffusion rate of extracellular MC-LR (0.7) was observed which was at the same level as co-culture (T 1) and significantly greater than the monoculture of the co-cultivation experiment (C 1) (*p* > 0.05 and *p* < 0.05, respectively, Fig. 4). As *Microcystis* cells were entering the death phase, the release of MC-LR increased due to the increased cell lysis (Fig. 1b) after 2 weeks of co-cultivation. Therefore, the MC was less distributed between the inner and outer side of the dialysis membrane (Figs 3 and 4).

## Discussion

In the present study, a co-cultivation system was designed using a dialysis membrane as a separation technique, where *Microcystis* strains were grown inside of the dialysis tubing and the green alga was cultured outside of the tubing. The system allowed the species to influence each other through their diffusible extracellular products without any physical cell-cell contacts. Thus, the shading effects of the buoyant *Microcystis* cells which were grown to a dense culture could be restricted through confining the *Microcystis* cells inside of the dialysis membrane in the mixed culture. The results showed that over time the extracellular MC-LR diffused through the membrane. However, the diffusion rate of the released MC across the membrane was affected by a sudden large release of extracellular MC-LR, possibly delaying diffusion to achieve an equilibrium on both sides.

The results showed that during the co-cultivation experiment, coinciding with the decreased cell density of *M. aeruginosa* toxic strain in both the control and treatments, the concentration of intracellular MC-LR remained unchanged in mono- and co-cultures. However, the total extracellular MC-LR from both the inside and outside was significantly higher, which can be explained by the *Microcystis* cells entering into the stationary phase of growth (Lyck 2004; Jähnichen et al. 2008). This means that *D. subspicatus* which was growing to the exponential phase of growth was exposed to the contents of the *Microcystis* cells that passed into the collapse phase of growth. The growth of the green alga was inhibited in co-cultivation with both toxic and non-toxic strains of *Microcystis*. However, MC-producing strain inhibited the growth of green alga greater and in earlier days, compared to the MC-deficient strain. Then, it was assumed that the toxic strain might benefit from MC production through outcompeting of the co-existing green alga. Moreover, the green alga showed a longer lag phase in the presence of MC-producing strain compared to the control and MC-deficient *Microcystis*. The study by Mohamed (2008) indicated that the green algae, *Chlorella* and *Scenedesmus*, could absorb and biotransform MCs. The inhibitory effects of MCs on the growth and photosynthesis of the other members of phytoplankton community have been reported in other investigations as well (Singh et al. 2001; Sukenik et al. 2002; Yang et al. 2014). MCs could negatively affect the exposed species through the restriction of the carbonic anhydrase activity (Sukenik et al. 2002), the reduction of CO_2_ uptake, depletion of nitrogen fixation (Singh et al. 2001), and the induction of oxidative stress that lead to programmed cell death (Pietsch et al. 2001; Pflugmacher 2004; Amado and Monserrat 2010). Therefore, it might be assumed that the green alga needed more time to fix the damages which might have resulted from the presence of MC and/or the other secondary metabolites of *Microcystis*.

The results indicated that the naturally occurring non-toxic strain of *M. aeruginosa* PCC 7005 negatively influenced the growth of green alga as well. However, it took longer, and the inhibition was significantly smaller than the co-cultures with the wild-type MC-producing strain PCC 7806. Then, the inhibitory effects of *Microcystis* on the growth of green alga was not only related to MC but also the increased release of the other probable secondary metabolites over time were involved. Previous studies showed the other secondary metabolites of *Microcystis* such as micropeptin, microviridin, microgenin, as well as some unidentified compounds might interfere in the interspecies interactions (Banker and Carmeli 1999; Reshef and Carmeli 2001; Ploutno et al. 2002). However, the results of the current study showed that the presence of MC might reinforce the inhibitory effects of *Microcystis* on the growth of the co-existing green alga. More studies need to be done concerning the analysis of the secondary metabolic profile of both MC-producing and -deficient strains, for undoubted confirm or rejection of the probable involvement of the other cyanobacterial metabolites in combination with or rather than MC in the interspecies interactions.

On the other hand, the green alga did not affect the MC production and release from MC-producing strain that might be related to the population ratio and the physiological status of the co-existing species which influenced the pattern of the algal communications. The study by Bittencourt-Oliveira et al. (2015) showed that at the equal initial population ratio (1:1, 1 × 10^5^ cells mL^−1^), *Scenedesmus acuminatus* induced MC production in *M. aeruginosa*. The study by Harel et al. (2013) showed that the interspecies interactions between *Scenedesmus huji* and *M. aeruginosa* spp. at the same initial density of 1 × 10^5^ cells mL^−1^, was depending on the physiological status of the species. They showed that the metabolites derived from the stationary phase of the growth of green alga, *S. huji*, caused severe cell lysis in *Microcystis* spp. through the decrease of the integrity of the cell membrane. The study by Yang et al. (2018) showed *M. aeruginosa* and *Scenedesmus obliquus* at the similar algal initial abundance of 1 × 10^5^ cells mL^−1^ have negatively affected one another’s growth at 20–30 °C, depending on their physiological status. As temperature increased from 20 to 30 °C, the competitive advantages of the green alga was decreased where the green alga was superior for a shorter time at the initial phase of co-cultivation while towards the end of the co-cultivation period *Microcystis* resumed dominance in the mixed culture. Moreover, the results indicated that the growth of MC-producing *Microcystis* was not influenced in the presence of the co-cultured green alga. However, the growth of MC-deficient strain was not monitored. Therefore, due to the lack of enough data it cannot be possible to assume whether the MC-producing strain benefited from MC over the MC-deficient strain to improve its fitness or not.

Taken together with the current study of the interspecies interplay between toxic and non-toxic strains of *M. aeruginosa* and the green alga, the probable importance of MC for the toxic *Microcystis* is evident. The results indicated that *Microcystis*, toxic and non-toxic strains, affected the growth of co-cultured green alga negatively. However, the growth inhibition in co-cultivation with the toxic *Microcystis* was significantly greater and occurred in earlier days. Then, in cyanobacterial blooms where toxic and non-toxic strains co-existed, the MC-producing strain may gain the advantage of MC production over non-toxic subpopulations to a greater exclusion of the co-existing species. MCs might play a role in the replacement of green algae with the cyanobacterial blooms at the end of summer.
